# Compact THz absorption spectroscopy using a LiNbO_3_ slot waveguide

**DOI:** 10.1038/s41377-025-02105-4

**Published:** 2026-01-04

**Authors:** Eric R. Sung, Keith A. Nelson

**Affiliations:** Department of Chemistry, Massachusetts Institute of Technology, Cambridge, MA USA

**Keywords:** Terahertz optics, Integrated optics, Imaging and sensing

## Abstract

THz spectroscopy is a powerful tool for studying a variety of samples, ranging from large biomolecules to solid-state materials. In cases where experimental space is limited or sample volumes are small, THz waveguides have been used to enable compact THz spectroscopy. The THz polaritonics platform is a waveguide-based approach that uses a thin lithium niobate slab to allow direct visualization of THz fields as they interact with structures integrated into the waveguide. Although there have been many successful studies using the platform for integrated photonics, the platform’s utility as a spectroscopic tool has been largely unexploited. Here, we use a slot waveguide integrated into the thin lithium niobate slab to measure the absorption spectrum of a sample inserted into the slot. The slot waveguide localizes the THz electric field within a low-index slot where a sample is placed. The THz fields propagate through the slot and are monitored as they interact with the sample. Perturbation theory is then used to extract the absorption spectrum and bulk refractive index of the sample with good sensitivity. These results show much promise for enabling compact linear and nonlinear THz spectroscopy using thin lithium niobate waveguides.

## Abstract (web summary)

We fabricate a lithium niobate slot waveguide for THz field enhancement and demonstrate compact linear THz spectroscopy on an inserted sample.

## Graphical abstract

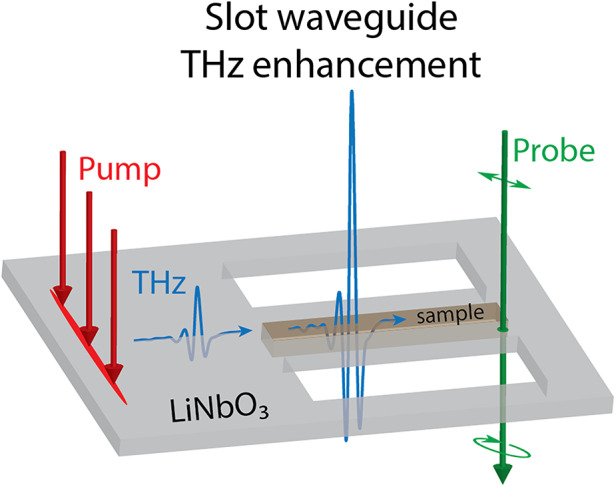

## Introduction

THz spectroscopy is a powerful tool with applications in chemical identification and non-destructive imaging^1,2^. Due to its relatively long wavelength and nonionizing nature (at 1 THz frequency, vacuum wavelength ${\lambda }_{0}=300 \, \upmu{\rm{m}}$ and photon energy = 4.1 meV), THz radiation is a good candidate for non-destructive imaging applications, including applications to biological samples^3,4^. In addition, many molecules have rotational or vibrational modes and many solid-state materials have collective modes in this frequency range, making it a good fingerprinting region for identifying and characterizing materials^5–7^. Numerous studies have used THz radiation to investigate solids^8,9^, liquid^10–12^, gases^13,14^, and recently, matter at extreme conditions^15,16^. Furthermore, THz spectroscopy has enabled detailed studies of dynamic systems, such as systems of hydrated proteins and large biomolecules^17,18^.

The THz polaritonics platform is a waveguide-based platform that uses a thin lithium niobate (LiNbO_3_, LN) or lithium tantalate (LiTaO_3_, LT) slab waveguide to enable compact THz experiments^19^. THz fields are generated directly within the LN or LT slab via optical rectification and are detected via electro-optic sampling performed in the waveguide. This platform has enabled many detailed studies of integrated photonics structures, such as photonic crystals^20–22^, cavities^23–25^, and many more^26^ during which the THz fields are directly monitored as they interact with the structures. However, the platform’s utility as an integrated spectroscopy platform to probe light-matter interactions has remained largely unexploited. Because the THz fields are generated in, confined to, and read out directly from the LN slab, the platform would be an appealing means to enable compact THz spectroscopy without the need for freely propagating THz fields and their associated bulky optics. However, the large THz refractive index of LN, results in limited interaction between the THz fields and a sample that would be deposited on the waveguide surface. Consequently, the THz signal resulting from the light-matter interaction may be very difficult to detect.

Various strategies have been employed to improve the sensitivity of waveguide-based spectroscopy in general. In the infrared frequency range, long interaction lengths and group-velocity engineering have been used to make the fields sensitive to dilute gases around the waveguide^27–29^. However, these methods typically require processing areas with dimensions roughly >100× larger than the target wavelength which makes scaling these techniques to THz frequencies difficult. An alternate strategy is to use plasmonic structures to localize an electromagnetic field in a small volume where the sample is placed. There have been several successful demonstrations^30–34^, but these studies relied on a somewhat narrow plasmonic resonance to target a specific frequency range around a known resonance in a sample. Using these techniques to investigate new materials with unknown absorption spectra would be time-consuming because only a small bandwidth could be interrogated at a time.

Here, we use a slot waveguide structure integrated into a thin LN slab in order to monitor the THz fields as they interact with a sample inserted into the slot over a relatively large bandwidth compared to other methods using enhancement structures. The slot waveguide consists of two thin strips of high-index material separated by a low-index slot^35,36^. When the THz field enters the slot waveguide, the electric field is enhanced in the low-index region. This allows for efficient coupling to a low-index sample inserted into the slot. By recording the THz fields as they propagate along the slot waveguide, we extract the refractive index and absorption spectrum of a sample inserted into the slot.

## Results

### Slot waveguide geometry

**Fig. 1:**
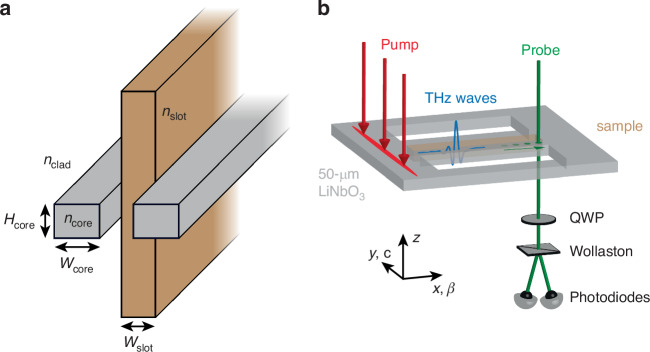
Slot waveguide geometry. **a** Schematic illustration of the slot waveguide structure. Two parallel strips of material with lateral dimensions ${W}_{\text{core}}$ and ${H}_{\text{core}}$ and refractive index ${n}_{\text{core}}$ are separated by a distance ${W}_{\text{slot}}$. A slab of sample material with refractive index ${n}_{\text{slot}}$ is inserted between the two strips and extends outside of the slot. The entire assembly is surrounded by cladding with refractive index ${n}_{\text{clad}}$. **b** Schematic illustration of the experimental geometry. The slot waveguide structure is fabricated in a 50 µm thick LN slab waveguide (*n*_core_ ≈ 5.1, *H*_core_ = 50 µm). A thin slab of sample is inserted into the slot. (The sample height along the *z*-axis is truncated for clarity.) The optical pump beam, polarized along the LN *c*-axis, is line-focused outside of the slot waveguide and launches a THz wave via optical rectification that propagates along the *x*-axis. The optical probe beam passes through one of the LN strips, followed by a quarter-wave plate (QWP) and a Wollaston prism. The probe intensity is measured using balanced photodiodes. The parameters used for the slot waveguide are: ${W}_{\text{core}}=50\,\upmu{\rm{m}}$, ${W}_{\text{slot}}=50\,\upmu{\rm{m}}$. The LN slab is free-standing and surrounded by air (${n}_{\text{clad}}=1$)

The slot waveguide is a structure used to localize the electric field in a low-index region over a rather large bandwidth^35,36^. The slot waveguide, shown in Fig. 1a, is composed of two parallel strips of high-index material with refractive index ${n}_{\text{core}}$ separated by a small sub-wavelength gap with width ${W}_{\text{slot}}$ and surrounded by a low-index cladding with refractive index ${n}_{\text{clad}} < {n}_{\text{core}}$. Maxwell’s equations require that for an electric displacement field ${\boldsymbol{D}}=\varepsilon {\boldsymbol{E}}$, where ${\boldsymbol{E}}$ is the electric field and $\varepsilon$ is the dielectric constant, the component of ${\boldsymbol{D}}$ that is polarized normal to a dielectric interface must be continuous when crossing the interface. Therefore, at the core/slot interface there is a discontinuity in ${\boldsymbol{E}}$ where the field strength jumps by a factor of ${n}_{\text{core}}^{2}/{n}_{\text{slot}}^{2}$, with the larger ${\boldsymbol{E}}$ field within the slot. This results in an enhanced electric field in the low-index slot along with increased sensitivity to a sample placed within the slot when ${n}_{\text{slot}} < {n}_{\text{core}}$. The experimental geometry, with the slot waveguide integrated into a thin LN slab, is shown in Fig. 1b.

The bandwidth over which we benefit from this enhancement can be roughly estimated as the regime where the THz half-wavelength in LN is longer than the dimensions of the LN strips on either side of the slot. In these experiments, we used ${n}_{\text{core}}\approx$5.1 and ${W}_{\text{core}}={H}_{\text{core}}=$ $50\,\upmu{\rm{m}}$, which means we get enhancement below ~0.6 THz. Furthermore, the enhancement is only present for samples where ${n}_{\text{slot}} < {n}_{\text{core}}$. For samples with refractive index approximately equal to the LN refractive index, the much simpler hybrid waveguide composed of thin slabs of LN and sample fixed together, such as the one used in ref. ^25^, results in sufficiently strong coupling between the THz fields and the sample. (See the SI for more details about the limitations of the hybrid waveguide geometry).

### Perturbation theory

In the weak absorption limit, material absorption can be described using perturbation theory^37^. (See the Supplementary Information for more details). Maxwell’s equations in the form of an eigenvalue problem can be written as

1 $$\nabla \times \nabla \times |{{\boldsymbol{E}}}_{n}\rangle ={\left(\frac{{\omega }_{n}}{c}\right)}^{2}\varepsilon |{{\boldsymbol{E}}}_{n}\rangle$$

where $\varepsilon$ is an operator for the dielectric permittivity map, $c$ is the vacuum speed of light, $|{{\boldsymbol{E}}}_{n}\rangle$ is the eigenmode describing the electric field profile, and ${\omega }_{n}$ is the angular eigenfrequency of the mode. If we add a small perturbation $\Delta \varepsilon$ to the permittivity map, the first-order correction to the eigenfrequency is calculated using

2 $${\omega }_{n}^{\left(1\right)}=-\frac{{\omega }_{n}^{\left(0\right)}}{2}\frac{\Delta \varepsilon }{\varepsilon }f$$

where ${\omega }_{n}^{\left(0\right)}$ is the unperturbed eigenfrequency and $f$ is the fill fraction defined as

3 $$f=\frac{{\langle {{\boldsymbol{E}}}_{n}^{(0)}|\varepsilon |{{\boldsymbol{E}}}_{n}^{(0)}\rangle }_{{V}_{{\rm{pert}}}}}{{\langle {{\boldsymbol{E}}}_{n}^{(0)}|\varepsilon |{{\boldsymbol{E}}}_{n}^{(0)}\rangle }_{V}}$$

where $|{{\boldsymbol{E}}}_{n}^{(0)}\rangle$ is the unperturbed electric field profile, the subscript $V$ denotes integration over all space, and ${V}_{\text{pert}}$ denotes integration only over the perturbed region. Physically, the fill fraction represents the fraction of the mode’s integrated electric energy density that is contained within the perturbed region and is a measure of how strongly the mode interacts with the perturbation.

Treating absorption as the perturbation (i.e., setting $\Delta \varepsilon$ equal to the imaginary part of the dielectric permittivity and $\varepsilon$ equal to the real part), the frequency-dependent effective absorption coefficient can be calculated as

4 $${\alpha }_{n}=-\frac{2{\omega }_{n}^{\left(1\right)}}{{v}_{{\rm{gr}}}}=\frac{{\omega }_{n}^{\left(0\right)}}{{v}_{{\rm{gr}}}}\frac{\Delta \varepsilon }{\varepsilon }f$$

where ${v}_{\text{gr}}$ is the group velocity of the unperturbed mode. The factor of 2 accounts for the fact that ${\alpha }_{n}$ describes how quickly the field intensity, rather than field amplitude, decreases.

### Experimental results

In order to measure THz absorption due to the inserted sample, the THz fields were measured along the LN strips with a test sample (*α*-lactose monohydrate) inserted and in a reference experiment using an empty slot waveguide. Space-time plots were constructed from the THz signals recorded at each position. Dispersion plots were calculated from the space-time plots through a 2D Fourier transformation, which transforms the time and space axes to frequency and wavevector axes, respectively.

**Fig. 2:**
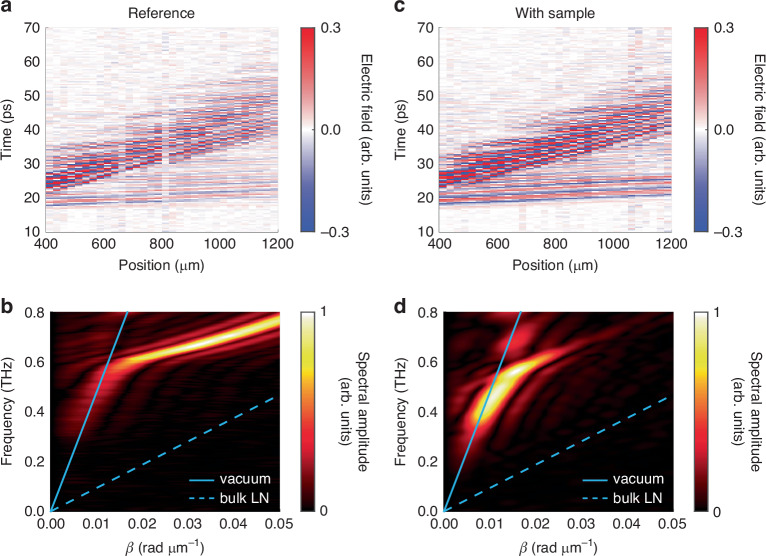
THz field propagation through the slot waveguide. **a**, **c** Representative space-time plots of the THz fields measured along the slot waveguides and **b**, **d** the corresponding dispersion plots. The reference measurements **a**, **b** were done with an empty slot waveguide. The plots in **c**, **d** show measurements with a lactose slab inserted into the slot. When calculating the dispersion plot in **d**, a window function was applied to the space-time plot in **c** to isolate the lower frequencies

Representative space-time plots showing the THz field propagation and dispersion plots are shown in Fig. 2. The reference space-time and dispersion plots are shown in Fig. 2a, b. The space-time plot shows a weak THz field with high group velocity and another stronger THz field with low group velocity. Although the space-time plot appears somewhat noisy, both THz signals are easily visible with a sufficient signal-to-noise ratio for useful measurements. (See the SI for more details.) The dispersion plot shows features that agree with these observations. Below 0.6 THz, the THz fields have nearly linear dispersion that approaches the vacuum light line. In this regime, the THz wavelength in LN (*λ* ≈ 100 µm at 0.6 THz) is significantly larger than the LN strip dimensions, so the core fill fraction is small and the effective refractive index for the mode is roughly 1. This is also consistent with the low THz signal strength because the electro-optic sampling signal is only sensitive to the THz field inside LN. Above 0.6 THz, the dispersion plot becomes much more intense and deviates significantly from the vacuum light line. In this regime, the THz wavelength becomes smaller than the LN strip dimensions and the effective refractive index correspondingly increases.

The space-time and dispersion plots for the slot waveguide with the lactose sample inserted are shown in Fig. 2c, d. The space-time plot shows features similar to those of the reference space-time plot. In order to better visualize the effect of the lactose slab, a window function was used to isolate the weak THz feature with higher group velocity in Fig. 2c when calculating the dispersion plot shown in Fig. 2d. A dip appears in the dispersion at around 0.53 THz, which matches the frequency of the well-known absorption peak in lactose^38,39^. In addition, the dispersion below 0.6 THz shifts to larger wavevectors corresponding to an increase in effective refractive index. This agrees with simulations of the experimental geometry, which are presented in the SI.

**Fig. 3:**
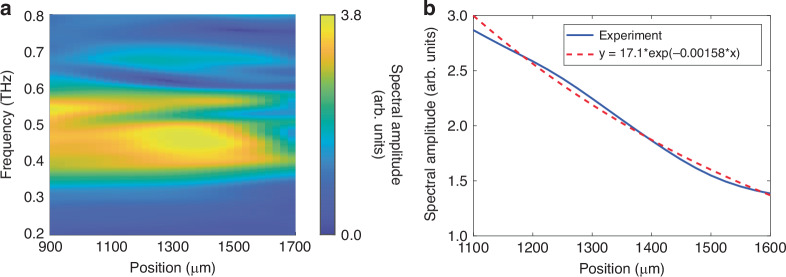
Spectral evolution with lactose inserted into the slot waveguide. **a** THz spectrum as a function of propagation distance. **b** A lineout of the plot in **a** taken at 0.53 THz (blue solid line). The data are fit to an exponential decay, which is overlaid (red dashed line)

The absorption spectrum of lactose can be determined by observing how the THz spectrum evolves as the THz field propagates through the slot waveguide. Note that, because the measurement window ends along the position axis before the THz signal completely disappears, the dispersion plots shown in Fig. 2b, d are artificially broadened due to spectral leakage. To avoid any resulting artifacts from affecting data analysis, we apply an inverse Fourier transform to the dispersion shown in Fig. 2d along the *β*-axis to convert the wavevector axis back to a position axis. Figure 3a shows the THz spectrum as a function of propagation distance. A clear absorption feature appears in the spectrum starting from around 1 mm into the filled slot region and becomes stronger as the propagation distance is increased. Note that in order to facilitate inserting the lactose slab into the slot, the lactose was inserted at a small angle. This results in part of the lactose slab hanging out of the slot, which effectively makes the slot empty at probe positions less than 1 mm. Figure 3b shows the spectral amplitude at 0.53 THz as a function of propagation distance within the filled slot region. There is a clear decrease in spectral amplitude that matches well with an exponential fit. From the fit, we read off the attenuation rate for the THz field amplitude as 15.8 cm^−1^. The effective absorption coefficient ${\alpha }_{\text{slot}}$ is defined as the rate at which the THz intensity (proportional to amplitude squared) decreases and is 31.6 cm^−1^. Note that because we monitor the THz fields as they propagate through the sample, the sample thickness along the THz propagation direction does not need to be measured unlike in traditional linear absorption spectroscopy.

## Discussion

### Calculating the bulk absorption coefficient

We can use perturbation theory to calculate the bulk absorption coefficient ${\alpha }_{\text{bulk}}$ from the experimental absorption coefficient ${\alpha }_{\text{slot}}$ for the sample inserted in the slot. Using a first-order calculation, the increase in absorption coefficient due to the sample is given by

5 $${\alpha }_{{\rm{slot}}}=\frac{\omega }{{v}_{{\rm{gr}},{\rm{slot}}}}\frac{{\varepsilon }_{{\rm{i}}}}{{\varepsilon }_{{\rm{r}}}}{f}_{{\rm{slot}}}$$

where $\omega$ is the THz angular frequency, ${v}_{{\rm{gr}},{\rm{slot}}}$ is the group velocity of the unperturbed slot waveguide, ${\varepsilon }_{\text{r}}$ is the unperturbed permittivity in the slot, and ${f}_{{\rm{slot}}}$ is the slot fill fraction. Here, we treat absorption, described by the imaginary part of the permittivity in the slot ${\varepsilon }_{\text{i}}$, as the perturbation. Similarly, the bulk absorption coefficient in the perturbative regime is given by

6 $${\alpha }_{{\rm{bulk}}}=\frac{\omega }{{v}_{{\rm{ph}},{\rm{bulk}}}}\frac{{\varepsilon }_{{\rm{i}}}}{{\varepsilon }_{{\rm{r}}}}$$

where ${v}_{{\rm{ph}},{\rm{bulk}}}$ is the bulk phase velocity, and ${\varepsilon }_{\text{r}}$ and ${\varepsilon }_{\text{i}}$ are the real and imaginary parts of the bulk permittivity, respectively. Combining these two equations, we get a formula to convert between the experimental absorption coefficient in the slot waveguide and the bulk absorption coefficient

7 $$\frac{{\alpha }_{{\rm{slot}}}}{{\alpha }_{{\rm{bulk}}}}=\frac{{v}_{{\rm{ph}},{\rm{bulk}}}}{{v}_{{\rm{gr}},{\rm{slot}}}}{f}_{\text{slot}}$$

The value for ${v}_{\text{gr},\text{slot}}$ can be determined from the slope of the experimental dispersion. Note that because we are only treating absorption as the perturbation, the group velocity should be taken for the slot waveguide structure with the sample inserted. From Fig. 2d, we estimate ${v}_{\text{gr},\text{slot}}=150\,\upmu{\rm{m}}/{\rm{ps}}$. The refractive index of lactose is ${n}_{\text{r}}=1.86$ ^40^, which gives bulk phase velocity ${v}_{\text{ph},\text{bulk}}=c/{n}_{\text{r}}=161\,\upmu{\rm{m}}/{\rm{ps}}$.

**Fig. 4:**
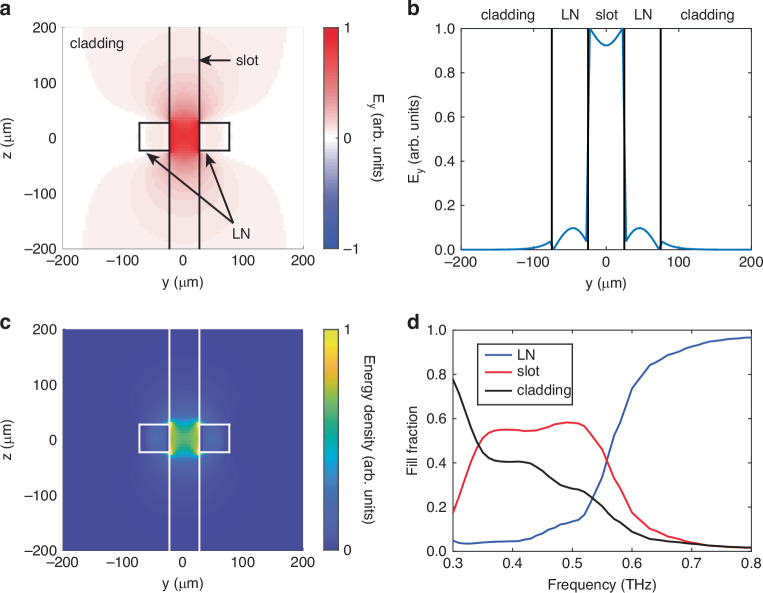
Fill fraction calculation. **a** Simulated ${E}_{y}$ field profile at 0.53 THz. The black lines indicate the dielectric interfaces of the loaded slot waveguide cross-section. The refractive index of the slot region was ${n}_{\text{slot}}$ = 1.86. Because the sample used in our experiments was effectively infinitely long along the *z*-axis, the slot region extended infinitely along the *z*-axis as opposed to only containing the region between the two LN strips. **b** THz field profile along a horizontal slice through the center of the structure (*z* = 0) extracted from **a**. The vertical black lines indicate the slot/LN and LN/cladding interfaces. **c** Calculated energy density profile for the EM mode shown in **a**. The white lines indicate the dielectric interfaces of the slot waveguide cross-section. All field components were included when calculating the energy distribution. **d** Calculated fill fractions in LN (blue), in the slot (red), and in the air cladding (black)

The fill fraction $f$ is calculated using the definition

8 $${f}_{\mathrm{slot}}=\frac{{\left\langle {{\boldsymbol{E}}}_{n}^{\left(0\right)}|{\varepsilon }_{{\rm{r}}}|{{\boldsymbol{E}}}_{n}^{\left(0\right)}\right\rangle }_{{v}_{\mathrm{slot}}}}{{\left\langle {{\boldsymbol{E}}}_{n}^{\left(0\right)}|{\varepsilon }_{{\rm{r}}}|{{\boldsymbol{E}}}_{n}^{\left(0\right)}\right\rangle }_{V}}$$

where $\left|{{\boldsymbol{E}}}_{n}^{\left(0\right)}\right\rangle$ is the electric field mode profile for the unperturbed structure and ${\varepsilon }_{\text{r}}$ is the (unperturbed) permittivity map. The subscripts denote integration over all space (*V*) and over the slot region (${V}_{\text{slot}}$). To determine $\left|{{\boldsymbol{E}}}_{n}^{\left(0\right)}\right\rangle$, we ran finite-difference time-domain EM simulations using the open-source software MEEP^41^. We used a monochromatic THz source and propagated the simulation until the transient signals disappeared. The slot waveguide only supports one guided mode with even symmetry at low frequencies <0.8 THz, so $\left|{{\boldsymbol{E}}}_{n}^{\left(0\right)}\right\rangle$ was taken simply as the electric field pattern at long times several wavelengths away from the source. Representative $y$-polarized THz electric field profiles are shown in Fig. 4a, b. Because we only treat absorption in the sample as the perturbation, the electric field profiles were calculated with the refractive index in the slot region set to the real refractive index of lactose (*n*_r_ = 1.86). Figure 4a shows clear localization of the electric field within the slot. A slice of the electric field profile through the middle of the structure (*z* = 0) is shown in Fig. 4b. Inside the high-index LN strips, the mode follows the typical sinusoidal spatial dependence, and in the low-index regions, the electric field decays exponentially. Due to the electric displacement continuity condition, there is a discontinuous jump in the electric field strength at each interface which results in a relatively low electric field strength in the LN strips. This is consistent with the weak THz signal observed in the experiment at lower frequencies. The calculated energy density of the slot waveguide mode is shown in Fig. 4c. Due to the large electric field strength in the slot, a significant portion of the energy density is localized within the slot, which results in efficient light-matter interactions. The calculated fill fractions in the slot and in the LN strips from 0.3 THz to 0.8 THz are shown in Fig. 4d. In the region between 0.35 THz and 0.50 THz, ${f}_{\text{slot}}$ is approximately 50%, indicating efficient coupling to the sample placed in the slot in that frequency range. On the other hand, the LN fill fraction ${f}_{\text{LN}}$ is quite low (<10%). Above 0.50 THz, ${f}_{\text{slot}}$ drops and ${f}_{\text{LN}}$ increases rapidly. This behavior is consistent with the experimental dispersion for the loaded slot waveguide (Fig. 2d) where the effective refractive index is nearly 1 at low frequencies and rapidly increases at frequencies above 0.6 THz. It is important to note that the large values for ${f}_{\text{slot}}$ below 0.6 THz prevent us from directly extracting the sample refractive index using first-order perturbation theory. In order for Eq. 2 to hold for a real-valued perturbation, we require $\frac{\Delta \varepsilon }{\varepsilon }{f}_{\text{slot}}\ll 1$. If we take an unloaded slot waveguide (*n*_slot_ = 1) as the unperturbed structure, then we require $\Delta \varepsilon \ll 2$, which would not be true for the vast majority of samples. Thus, ${f}_{\text{slot}}$ must be calculated with the sample already inserted into the slot.

In order to calculate the bulk absorption coefficient, we return to Eq. 7 and plug in the corresponding values: ${v}_{\text{ph},\text{bulk}}=161\,\upmu{\rm{m}}/{\rm{ps}}$, ${v}_{\text{gr},\text{slot}}=150\,\upmu{\rm{m}}/{\rm{ps}}$, ${f}_{{slot}}=0.537\times 0.8=0.427$. Note that the values for ${f}_{\text{slot}}$ in Fig. 4d should be multiplied by 0.8 to account for the fact that the slot is 50 µm wide while the inserted lactose slab is only 40 µm wide. (See SI for a more detailed calculation). This gives a peak bulk absorption coefficient ${\alpha }_{\text{bulk}}=67\,{{\rm{cm}}}^{-1}$, which agrees very well with the literature value *α* = 66.84 cm^−1^ ^40^.

### Extraction of bulk material parameters

**Fig. 5:**
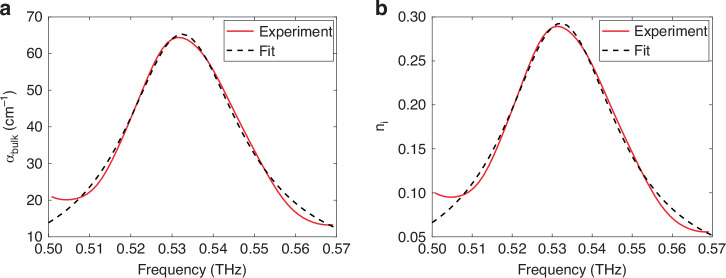
Extracting parameters for bulk lactose. **a** Values for ${\alpha }_{\text{bulk}}$ calculated using Eq. 7. **b** Values for imaginary refractive index ${n}_{\text{i}}$ calculated using Eq. 9. The Lorentzian fit (dashed line) using the parameters output by the iterative procedure is also plotted in **a** and **b**

Figure 5a shows the calculated values for ${\alpha }_{\text{bulk}}$ for a range of frequencies around 0.53 THz. ${\alpha }_{\text{bulk}}$ can be converted to an imaginary refractive index ${n}_{\text{i}}$ using the relationship

9 $${\alpha }_{{\rm{bulk}}}=\frac{{4\pi n}_{{\rm{i}}}}{{\lambda }_{0}}$$

where ${\lambda }_{0}$ is the vacuum wavelength. The calculated values for ${n}_{\text{i}}$ are shown in Fig. 5b. We can fit ${n}_{\text{i}}$ to the Lorentzian oscillator model using the equation

10 $$\varepsilon \left(\omega \right)={\left[{n}_{{\rm{r}}}\left(\omega \right)+{{in}}_{{\rm{i}}}\left(\omega \right)\right]}^{2}={\varepsilon }_{{\infty }}+\frac{\sigma {\omega }_{0}^{2}}{{\omega }_{0}^{2}-{\omega }^{2}+i\omega \Gamma }$$

where ${\varepsilon }_{{\infty }}$ is the high-frequency permittivity, ${\omega }_{0}$ is the resonance frequency of the oscillator, $\Gamma$ is the damping rate, and $\sigma$ is the oscillator strength. The extracted values are given in Table 1. The fit recovers values that agree quite well with literature values, which also are tabulated for comparison.

**Table 1:** Extracted material parameters for lactose using a LN slot waveguide

	Extracted values (using literature ${\varepsilon }_{{\infty }}$)	Extracted values (using iterative procedure)	Literature values
${\varepsilon }_{{\infty }}$	3.35*	3.3	3.35
$\sigma$	0.054	0.055	0.0524
${\omega }_{0}/2\pi$ (THz)	0.53	0.53	0.5303
$\Gamma /2\pi$ (GHz)	34	34	25.8

Literature values taken from ref. ^40^ * indicates that the parameter was fixed to this value during fitting

It should be noted that lactose is a well-studied material in the THz frequency range, so the material parameters (notably the high-frequency permittivity ${\varepsilon }_{{\infty }}$) are readily available and could be used for the calculation. For a novel material, ${\varepsilon }_{{\infty }}$ is not necessarily known beforehand so there would be no value for ${v}_{\text{ph},\text{bulk}}$ to use in Eq. 7 to calculate ${\alpha }_{\text{bulk}}$ from ${\alpha }_{\text{slot}}$. Furthermore, recall that only the imaginary part of the sample permittivity is the perturbation, so the value of ${f}_{\text{slot}}$, which depends on the sample’s refractive index, is also uncertain. To extract ${\varepsilon }_{{\infty }}$ without prior knowledge, we use the following iterative process.

1. Choose an initial guess ${\varepsilon }_{{\infty }}^{\left(1\right)} > 1$.
2. Calculate $${\alpha }_{\text{bulk}}$$ using $${v}_{\text{ph},\text{bulk}}=c/\sqrt{{\varepsilon }_{{\infty }}}$$.
3. Calculate ${n}_{\text{i}}$ from ${\alpha }_{\text{bulk}}$ using Eq. 7 and fit to Eq. 10.
4. Calculate the refined value ${\varepsilon }_{{\infty }}^{\left(n+1\right)}$ as the average value between ${\varepsilon }_{{\infty }}^{\left(n\right)}$ and the value for ${\varepsilon }_{{\infty }}$ output by the fit. During this step, ${f}_{\text{slot}}$ is also recalculated based on the updated ${\varepsilon }_{{\infty }}^{\left(n+1\right)}$.
5. Repeat steps 2–4 until ${\varepsilon }_{{\infty }}^{\left(n\right)}$ converges with the output from the fit.

Starting with an initial guess ${\varepsilon }_{{\infty }}^{\left(1\right)}=2.0\,({n}_{\text{r}}=1.4,{v}_{\text{ph}},{\text{bulk}}=210\,\upmu{\rm{m}}/{\rm{ps}})$, this procedure converges after 5 iterations to ${\varepsilon }_{{\infty }}=3.3$, which agrees well with the literature value. The material parameters output by the iterative procedure are given in Table 1.

Here we use a slot waveguide to localize the THz electric field inside a low-index region for improved coupling to a sample. A thin slab of *α*-lactose monohydrate was inserted into the slot and the absorption spectrum was measured with good sensitivity over a relatively large bandwidth for methods that use enhancement structures. The bulk absorption spectrum was calculated using perturbation theory and fit to a Lorentzian oscillator model to yield material parameters that agree very well with literature. A fitting procedure that does not assume any prior knowledge about the sample is also presented, which could be used to characterize novel materials. This work represents, to our knowledge, the first demonstration of dielectric slot waveguides for spectroscopy at THz frequencies.

The usable THz bandwidth can be further increased by using a thinner LN slab (decreasing ${H}_{\text{core}}$ in Fig. 1a) or by decreasing the width of the LN strips (${W}_{\text{core}}$ in Fig. 1a). In this case, the LN slab would be more fragile but could be glued to a glass slide for support. Another potential way to increase the bandwidth would be to include the higher-order slot waveguide modes in the analysis. In general, the higher-order modes should provide good enhancement at some higher-frequency regions. However, they will interfere with the lowest-order mode used here and also have lower detection efficiency because of the more rapidly oscillating THz electric field in LN, so the modes would need to be separated and treated carefully. Greater sensitivity could be achieved using slow-light engineering as is done with photonic crystal slot waveguides^27^. Doing so would limit the probed bandwidth and would require significantly longer times to pattern the waveguide, but could potentially be used to focus on a smaller spectral range of interest identified when using the slot waveguide presented in this work.

Our results open up a lot of potential for compact THz spectroscopy. Although this method probes a smaller spectral bandwidth and has lower SNR compared to traditional THz time-domain spectroscopy (THz-TDS), its waveguide-based nature brings several advantages. In the method presented here, THz generation, propagation, and detection all occur directly within the LN waveguide. This could allow THz experiments to be performed in situations where space is limited by removing the need for bulky optics typically used in a free-space THz setup. Most other waveguide-based methods also generate and detect the THz fields outside of the waveguide, so some THz optics, and consequently more space for the experimental setup, would still be required. The method presented here could be especially useful in cases where specialized sample environments make it difficult to use a traditional THz-TDS setup, e.g., samples located inside an XFEL chamber or deep within the bore of a cryomagnet. Another advantage is that smaller sample volumes are needed. In our experiments, the sample was a 1.5 mm × 500 µm × 40 µm slab. In traditional THz-TDS experiments, the sample is frequently a ~ 1 mm thick pressed pellet with a several mm^2^ cross-sectional area to accommodate the large THz focus (typically a few hundred microns to few millimeters across). One aspect unique to this method is that our measurement of the absorption spectrum does not require knowledge of the sample thickness. Typical THz-TDS experiments require an external measurement of the sample thickness to accurately determine material properties such as refractive index or absorption coefficient. In our method, the THz fields are monitored as they propagate through the slot waveguide, which effectively provides multiple measurements at various sample thicknesses.

Although this work focuses on linear THz spectroscopy, these results also could facilitate compact nonlinear THz spectroscopy. Recent work has increased the field levels achievable in thin LN and lithium tantalate waveguides^42,43^, reaching the low 100 kV/cm levels where many materials start showing nonlinear behavior^44–46^. A slot waveguide could be used to localize these strong THz fields within a sample inserted into the slot, which could enable compact nonlinear THz spectroscopic measurements. This is particularly appealing for enabling nonlinear spectroscopic studies in the specialized sample environments mentioned earlier, where traditional high-field THz generation methods (e.g., tilted-pulse front) bring further complications to the experimental design, such as large space requirements or relying on noncollinear geometries, adding more constraints that make the experiment very difficult to execute.

## Materials and methods

### Slot waveguide fabrication and loading

The slot waveguide was fabricated in a 50-µm LN slab waveguide (NanoLN) using chemically-assisted femtosecond laser machining^47^. The slot and LN strips were 50 µm wide (*W*_slot_ = 50 µm, *W*_core_ = 50 µm). The entire slot waveguide structure was 2.5 mm long.

As a test sample, we used *α*-lactose monohydrate (Sigma-Aldrich, 99% purity, CAS 5989-81-1) due to its strong, sharp absorption peak centered at 0.53 THz which corresponds to a hindered rotation of the lactose molecules in the crystal^38,39^. The lactose was dissolved in deionized water to make a saturated solution. The solution was then poured into a crystallizing dish and the solvent was allowed to evaporate under ambient conditions to form a ~500 µm thick layer of polycrystalline lactose. Femtosecond laser machining was used to cut a 40 µm by 1.5 mm slice of the lactose. The lactose slab was purposely cut to be slightly smaller than ${W}_{\text{slot}}$ to facilitate inserting the slab into the slot. To load the sample into the slot, the thin lactose slab was placed on a stage with manual *x*,*y*,*z* control. The stage was aligned to the slot and the lactose slab was then gently pushed into the slot using a razor blade.

### Experimental setup

The experiments were performed using the output from a Ti:sapphire laser (center wavelength = 800 nm, pulse duration = 100 fs, repetition rate = 1 kHz). The output was split in a 95:5 ratio into the pump and probe beams, which entered the LN slab from opposite sides. The pump pulses were attenuated to 200 µJ and focused onto the LN slab to a line 100 µm away from the edge of the slot waveguide using a 25-cm cylindrical lens. A dichroic mirror diverted the focusing pump beam onto the LN slab and was used to help separate the pump beam and the 400-nm probe beam described later. The pump was linearly polarized along the LN *c*-axis (*y*-axis in Fig. 1b), which resulted in THz generation via optical rectification directly within the waveguide. Due to the large difference in LN refractive index at optical and THz frequencies, the resulting THz field is launched with a significant lateral wavevector component and couples into the TE dielectric waveguide modes. An optical chopper was used to modulate the pump beam at 500 Hz. The THz fields were measured directly within the LN waveguide using methods described in ref. ^48^ The probe beam was frequency-doubled using a *β*-barium borate (BBO) crystal. The 400-nm output from the BBO was linearly polarized 45° relative to the LN optic axis and was focused into one of the LN strips in the slot waveguide using a 15-cm lens. The probe beam then passed through the dichroic mirror for directing the pump beam onto the LN slab, followed by a quarter-wave plate and Wollaston prism and the intensities of the two polarization components were measured using balanced photodiodes. A BG40 colored glass filter was used to spectrally filter out any scattered 800-nm pump light that reached the photodiodes. A delay stage was used to control the pump-probe delay. The probe beam was stepped along the slot waveguide in 25-µm increments. This was done by mounting the LN slot waveguide on a motorized translation stage, which moved the slot waveguide along the *x*-direction indicated in Fig. 1b. The final focusing lens and dichroic mirror for the pump beam were placed on a second motorized translation stage and were moved to reposition the pump beam on the same pumping region each time the slot waveguide was moved. The change in pump arrival time was compensated using the pump-probe delay stage. For the reference experiments, the probe started 100 µm away from the edge of the slot waveguide (200 µm away from the pump). For the experiments with lactose inserted, the probe started at the edge of the lactose slab (225 µm into the slot waveguide; 325 µm away from the pump) and scanned over the extent of the filled region.

## Supplementary information

Supplementary Information for Compact THz absorption spectroscopy using a LiNbO_3_ slot waveguide

## Data Availability

Data underlying the results presented in this paper are not publicly available at this time but may be obtained from the authors upon reasonable request.
