# Supplementary material for: Compact THz absorption spectroscopy using a LiNbO3 slot waveguide
Source: Light Sci Appl. 2026 Jan 4;15:47. doi: 10.1038/s41377-025-02105-4 (PMC12764560; doi:10.1038/s41377-025-02105-4)
Supplement: Supplementary file 1 — Supplementary Information for Compact THz absorption spectroscopy using a LiNbO3 slot waveguide [file 41377_2025_2105_MOESM1_ESM.pdf]

Supplementary Information for Compact THz absorption spectroscopy using a $LiNbO_3$ slot waveguide

Eric R. Sung[1], Keith A. Nelson[1]*

[1]*Department of Chemistry, Massachusetts Institute of Technology, Cambridge, MA, USA 02139*

Contents:

1. Dielectric slab waveguide modes
2. Slot waveguide dispersion
3. Image of sample inserted into slot
4. Signal-to-noise comparison
5. Perturbation theory
6. FDTD simulations
7. Effect of air gap
8. References

S.1 Dielectric slab waveguide modes

Because the thickness of an unpatterned 50-μm LN slab is on the order of the THz wavelength, it acts as a dielectric waveguide and the THz fields propagate as dielectric waveguide modes with modified dispersion. A full description is given in Reference [1]. The THz fields are excited with in-plane polarization, corresponding to excitation of the transverse electric (TE) waveguide modes. Fig. S1 shows the three lowest order TE waveguide mode profiles, their dispersion, and calculated cladding fill fractions.

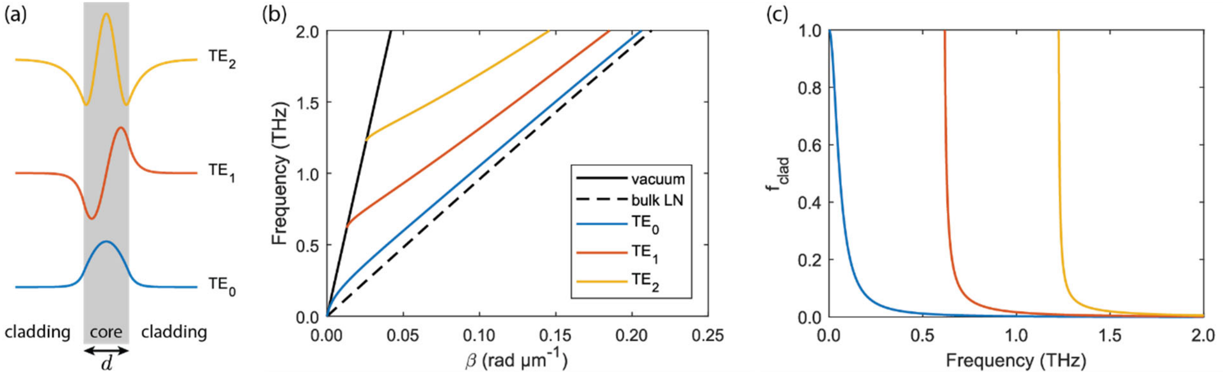

Figure S1. Dielectric slab waveguide. (a) Electric field profiles for the TE_0, TE_1, and TE_2 dielectric waveguide mode profiles at 1.5 THz. (b) Calculated TE waveguide dispersion curves for a LN slab (n_e = 5.1) with thickness d = 50 μm. (c) Calculated cladding fill fractions.

The cladding fill fraction is quite small over most of the THz bandwidth. This results in very limited interaction between the waveguided THz field and the surrounding medium. Likewise, interactions between the THz field and a sample deposited on the surface of the waveguide would also be limited. Strong interactions between the THz field and an adjacent sample are possible when the sample refractive index approximately matches the LN refractive index, like in the hybrid waveguide geometry used in Reference [2], in which case the LN and sample together effectively act like a thicker dielectric slab.

S.2 Slot waveguide dispersion

To our knowledge, there is no analytical solution for the dispersion curves for the 2D slot waveguide used in this work. However, the 1D slot waveguide (taken in the limit $H_{\text{core}} \to \infty$) can be solved analytically. While it is not exactly the same system, we can gain intuition for the 2D slot waveguide by treating the 1D case.

The electric field profile for the even-symmetry TM eigenmodes of the 1D slot waveguide (analogous to the even-symmetry TE-like eigenmodes of the 2D slot waveguide that we excite in the experiment) can be calculated using Maxwell's equations. The transversality constraint for the TM modes requires that the electric displacement field $\boldsymbol{D} = \varepsilon \boldsymbol{E}$ be continuous across each interface. The solution, given in Reference [3], is:

$$E_x(x) = E_0 \begin{cases} \frac{1}{n_{\text{slot}}^2}\cosh(\gamma_{\text{slot}}x) & |x| \le \frac{W_{\text{slot}}}{2} \\ \frac{1}{n_{\text{core}}^2}\left\{\cosh\left(\frac{\gamma_{\text{slot}}W_{\text{slot}}}{2}\right)\cos\left[k_{\text{core}}\left(|x|-\frac{W_{\text{slot}}}{2}\right)\right] + \frac{n_{\text{core}}^2\gamma_{\text{slot}}}{n_{\text{slot}}^2k_{\text{core}}}\sinh\left[\frac{\gamma_{\text{slot}}W_{\text{slot}}}{2}\right]\sin\left[k_{\text{core}}\left(|x|-\frac{W_{\text{slot}}}{2}\right)\right]\right\} & \frac{W_{\text{slot}}}{2} < |x| \le \frac{W_{\text{slot}}}{2} + W_{\text{core}} \\ \frac{1}{n_{\text{clad}}^2}\left\{\cosh\left(\frac{\gamma_{\text{slot}}W_{\text{slot}}}{2}\right)\cos[k_{\text{core}}W_{\text{core}}] + \frac{n_{\text{core}}^2\gamma_{\text{slot}}}{n_{\text{slot}}^2k_{\text{core}}}\sinh\left[\frac{\gamma_{\text{slot}}W_{\text{slot}}}{2}\right]\sin[k_{\text{core}}W_{\text{core}}]\exp\left[-\gamma_{\text{clad}}\left(|x|-\left(\frac{W_{\text{slot}}}{2}+W_{\text{core}}\right)\right)\right]\right\} & \frac{W_{\text{slot}}}{2} + W_{\text{core}} < |x| \end{cases}$$

where E_0 is an arbitrary constant that scales the overall amplitude of the mode, k_{core} is the transverse wavenumber in the high-index core, and γ_{slot} and γ_{clad} are the field decay coefficients in the slot and cladding regions, respectively. The refractive indices (n_{slot}, n_{core}, n_{clad}) and waveguide dimensions (W_{slot}, W_{core}) are defined in Fig. 1a in the main text. The parameters k_{core}, γ_{slot}, and γ_{clad} obey the relations

$$k_0^2n_{\text{core}}^2 - k_{\text{core}}^2 = k_0^2n_{\text{slot}}^2 + \gamma_{\text{slot}}^2 = k_0^2n_{\text{clad}}^2 + \gamma_{\text{clad}}^2 = \beta^2$$

where k_0 is the vacuum wavenumber and β is the propagation constant for the eigenmode. To solve for β, we numerically solve the characteristic equation

$$\tan\left[k_{\text{core}}W_{\text{core}} - \arctan\left(\frac{n_{\text{core}}^2\gamma_{\text{clad}}}{n_{\text{clad}}^2k_{\text{core}}}\right)\right] = \frac{n_{\text{core}}^2\gamma_{\text{slot}}}{n_{\text{slot}}^2k_{\text{core}}}\tanh\left(\frac{\gamma_{\text{slot}}W_{\text{slot}}}{2}\right)$$

Assuming an air cladding, ($n_{\text{slot}} \ge n_{\text{clad}}$), in order to have a bounded mode, β is constrained by

$$k_0n_{\text{slot}} < \beta < k_0n_{\text{core}}$$

The cutoff frequency f_{cutoff} can be found by solving the characteristic equation at the minimum value for β, i.e. when $\beta = k_0n_{\text{slot}} \rightarrow \gamma_{slot} = 0$.

$$f_{m,\text{cutoff}} = \frac{c}{2\pi W_{\text{core}}\sqrt{n_{\text{core}}^2 - n_{\text{slot}}^2}}\left[m\pi + \arctan\left(\frac{n_{\text{core}}^2}{n_{\text{clad}}^2}\sqrt{\frac{n_{\text{slot}}^2 - n_{\text{clad}}^2}{n_{\text{core}}^2 - n_{\text{slot}}^2}}\right)\right]$$

where c is the vacuum speed of light and m is an integer for enumerating the modes. In an unloaded slot waveguide, we have $n_{\text{slot}} = n_{\text{clad}}$ and the $\arctan(\cdot)$ term disappears. As a result, the TM_0 mode has zero cutoff frequency. When a sample is loaded, we generally have $n_{\text{slot}} > n_{\text{clad}}$ and the $\arctan(\cdot)$ term increases the cutoff frequency for all modes.

Fig. S2 shows the calculated 1D slot waveguide dispersion and fill fractions for both the unloaded (n_{slot} = 1.0) and loaded (n_{slot} = 1.86) cases. The widths of the LN strips (W_{core}) and slot (W_{slot}) were 50 µm. When the slot is loaded, the dispersion shifts to higher wavevectors. In addition, the bend in the dispersion at around 0.6 THz becomes less pronounced.

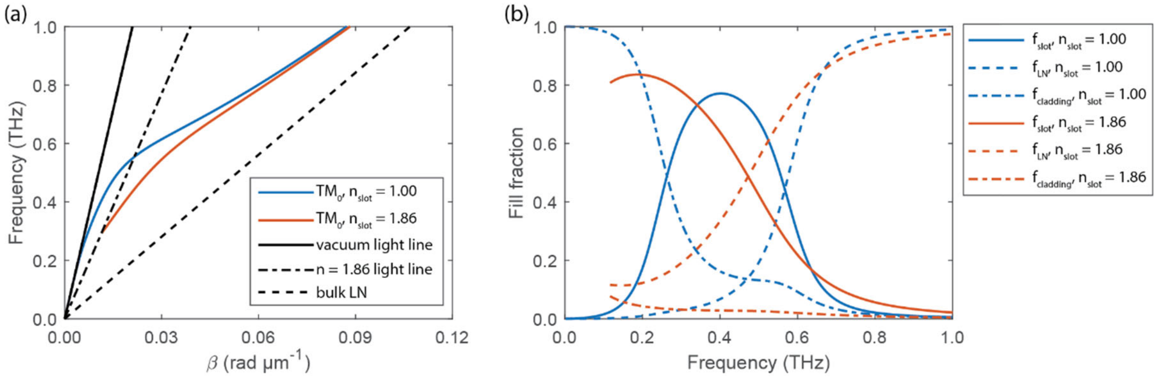

Figure S2. 1D slot waveguide. (a) Calculated TM waveguide dispersion curves and (b) calculated fill fractions for the 1D slot waveguide.

In order to verify that the 2D slot waveguide shows similar behavior, we ran finite-difference time-domain simulations of the 2D slot waveguide. A general description of how the simulations were performed is provided in Section S.6. We used a broadband source to excite a large frequency range, then calculated the THz electric field as a function of time and propagation distance. The dispersion was calculated via a 2D Fourier transformation, converting the time and space axes to frequency and wavevector axes, respectively, and is plotted in Fig. S3. The calculated dispersion for the 2D slot waveguide shows similar qualitative behavior as the 1D slot waveguide, namely that insertion of a sample pushes the dispersion to higher wavenumbers and smooths out the bend at 0.6 THz. Furthermore, these calculated dispersion plots show good agreement with the experimental dispersion plots shown in Fig. 2 in the main text.

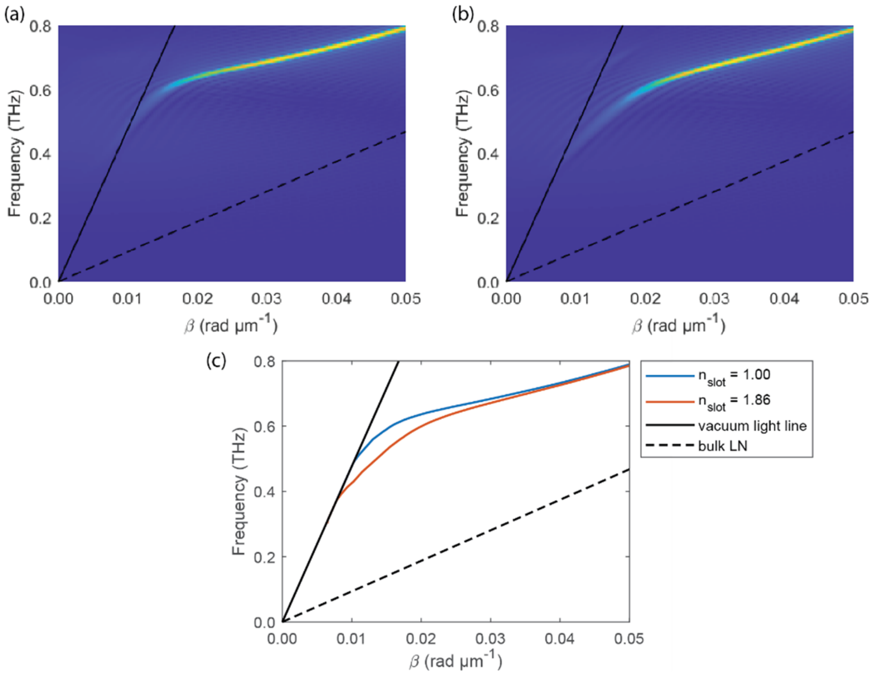

Figure S3. 2D slot waveguide. Calculated dispersion for (a) an empty 2D slot waveguide and (b) 2D slot waveguide loaded with a sample with n_{slot} = 1.86. The dimensions of the slot waveguide were the same as the ones used in the experiment. (c) Slot waveguide dispersion curves extracted from (a) and (b), plotted together for comparison.

S.3 Image of sample inserted into slot

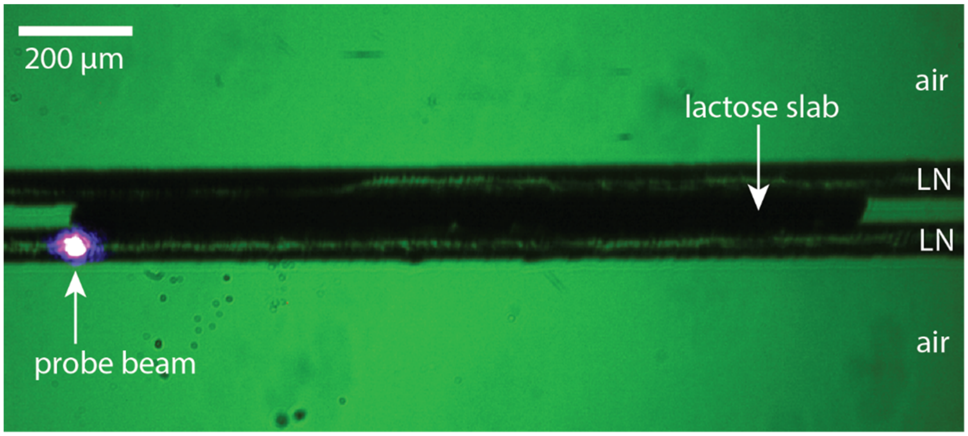

Figure S4. Optical image of the sample assembly. Optical image of the slot waveguide loaded with a lactose slab taken in the experimental setup. An LED was used to back-illuminate the filled slot waveguide and the transmitted light was recorded using a CMOS camera. The optical probe beam used to detect the THz fields can also be seen in the image.

S.4 Signal-to-noise comparison

The slot waveguide functions by localizing the THz field within the slot region. However, in our experiments, we rely on the electro-optic effect in LN in order to monitor the THz field as it propagates through the slot waveguide. As a result, the THz field strength we detect in the slot waveguide is smaller than what we would observe in an unpatterned waveguide. Fig. S5 shows representative THz traces recorded in the slot waveguide and in an unpatterned 50-μm LN waveguide typically used in polaritonics experiments. In both cases, the THz signal is recorded after 100 μm of propagation through the waveguide. To compare the two cases, we compute the signal-to-noise ratio (SNR) by dividing the peak THz field amplitude by the noise observed in the time trace before the THz pulse arrives. The SNR for the unpatterned waveguide is 650. The SNR for the THz signal in the slot waveguide is 56. For the weaker, faster-moving THz signal where we get enhancement from the slot waveguide, the SNR is about 20, which is quite a bit lower than the SNR in the reference unpatterned waveguide. However, the signal is still more than an order of magnitude larger than noise, indicating that we can still reliably monitor the THz signal in the slot waveguide despite the THz field being mostly localized within the slot. Additionally, the analysis used in the main text relies on comparing multiple THz spectra recorded at different positions along the slot waveguide, which helps suppress the effect of random noise.

Part of the decrease in SNR in the slot waveguide is due to imperfections in the slot waveguide during fabrication (visible as the black textures on the LN strips in Fig. S4). The SNR could be improved if the imperfections are minimized, potentially through careful shaping of the laser pulses used for machining [4].

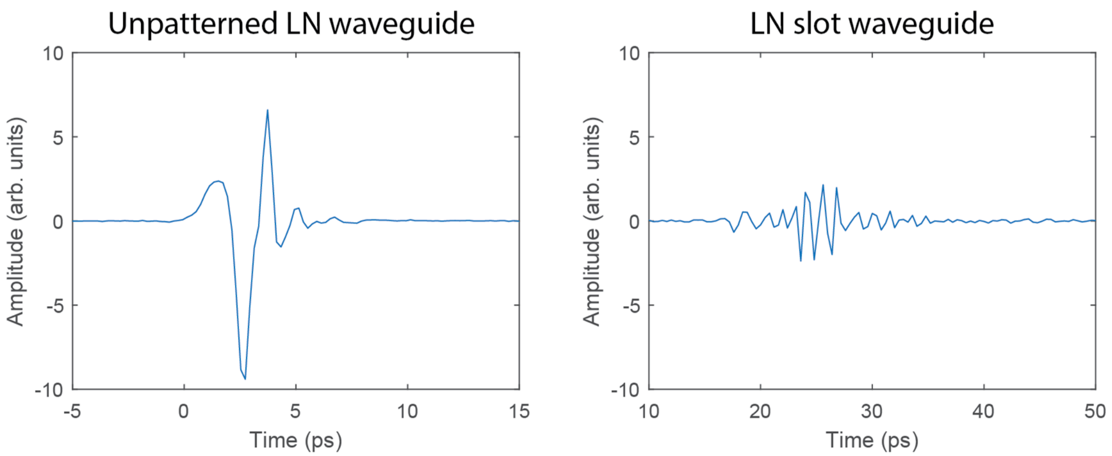

Figure S5. SNR comparison. Representative time-domain THz signals recorded in (left) an unpatterned 50-μm LN waveguide (right) and the LN slot waveguide used in the experiments.

S.5 Perturbation theory

Perturbation theory when applied to Maxwell's equations is a powerful tool for modeling light-matter interactions, such as absorption, gain, and weak nonlinearities [5]. Maxwell's equations written as an eigenproblem for the electric field $|\boldsymbol{E}\rangle$ is given by

$$\nabla \times \nabla \times |\boldsymbol{E}_n\rangle = \left(\frac{\omega_n}{c}\right)^2 \varepsilon |\boldsymbol{E}_n\rangle$$

where ω_n is the angular frequency of the nth electromagnetic mode $|\boldsymbol{E}_n\rangle$, c is the vacuum speed of light, and ε is a map of the dielectric permittivity. Here, we use Dirac notation to express the orthonormal basis set of electromagnetic modes $\left|\boldsymbol{E}_n^{(0)}\right\rangle$ subject to the orthonormality condition $\left\langle \boldsymbol{E}_n^{(0)} \middle| \varepsilon \middle| \boldsymbol{E}_{\boldsymbol{m}}^{(0)} \right\rangle = \delta_{nm}$. To apply perturbation theory, we add a small change in permittivity $\Delta\varepsilon$ to the dielectric map and expand the eigenfrequencies and eigenmodes.

$$\varepsilon \to \varepsilon + \Delta\varepsilon$$
$$|\boldsymbol{E}_n\rangle \to \left|\boldsymbol{E}_n^{(0)}\right\rangle + \left|\boldsymbol{E}_n^{(1)}\right\rangle$$
$$\omega_n \to \omega_n^{(0)} + \omega_n^{(1)}$$

Here, the superscript $^{(0)}$ denotes the unperturbed value and $^{(1)}$ denotes the first-order correction. After making these substitutions, collecting terms to first order, and left multiplying by the mode $\left\langle \boldsymbol{E}_n^{(0)} \right|$, we get the equation

$$\omega_n^{(1)} = -\frac{\omega_n^{(0)}}{2} \frac{\left\langle \boldsymbol{E}_n^{(0)} \middle| \Delta\varepsilon \middle| \boldsymbol{E}_n^{(0)} \right\rangle}{\left\langle \boldsymbol{E}_n^{(0)} \middle| \varepsilon \middle| \boldsymbol{E}_n^{(0)} \right\rangle}$$

We can define the fill fraction f as the integral

$$f = \frac{\left\langle \boldsymbol{E}_n^{(0)} \middle| \varepsilon \middle| \boldsymbol{E}_n^{(0)} \right\rangle_{V_{\mathrm{pert}}}}{\left\langle \boldsymbol{E}_n^{(0)} \middle| \varepsilon \middle| \boldsymbol{E}_n^{(0)} \right\rangle_{V}}$$

where the subscript V denotes integrating over all space and V_{pert} denotes integrating only over the perturbed region. Assuming that ε and $\Delta\varepsilon$ are both scalars over the perturbed region, we get the equation

$$\omega_n^{(1)} = -\frac{\omega_n^{(0)}}{2}\frac{\Delta\varepsilon}{\varepsilon}f$$

The first-order correction to frequency can be converted to a first-order correction to propagation constant $\beta_n^{(1)}$ by dividing by the group velocity v_{gr}.

$$\beta_n^{(1)} = -\frac{\omega_n^{(1)}}{v_{\mathrm{gr}}} = \frac{\omega_n^{(0)}}{2v_{\mathrm{gr}}}\frac{\Delta\varepsilon}{\varepsilon}f$$

Note that the Cauchy-Reimann equations guarantee that this equation applies for both real-valued and imaginary-valued perturbations. Therefore, by treating the imaginary part of the permittivity map as the perturbation, we can treat material absorption. If we take the real part of the permittivity as the unperturbed permittivity map and the imaginary part as the perturbation (i.e. $\varepsilon = \varepsilon_{\mathrm{r}}$ and $\Delta\varepsilon = \varepsilon_{\mathrm{i}}$), the absorption coefficient can be calculated using

$$\alpha_n = 2\beta_n^{(1)} = \frac{\omega_n^{(0)}}{v_{\mathrm{gr}}}\frac{\varepsilon_{\mathrm{i}}}{\varepsilon_{\mathrm{r}}}f$$

The factor of 2 comes from the fact that the absorption coefficient α_n describes the rate at which the electric field intensity decreases whereas $\beta_n^{(1)}$ describes the rate at which the electric field amplitude decreases. For a bulk material, we set f equal to 1. We can also make the approximation that the group velocity equals the phase velocity v_{ph}. This is because setting the imaginary part of the unperturbed permittivity to 0 requires that the real part be a constant to fulfill the Kramers-Kronig relations. Making these substitutions gives the equation

$$\alpha_n = \frac{\omega_n^{(0)}}{v_{\mathrm{ph}}}\frac{\varepsilon_{\mathrm{i}}}{\varepsilon_{\mathrm{r}}}$$

This equation can be manipulated to recover the textbook definition for the absorption coefficient. We relate the real and imaginary parts of the refractive index (n_{r} and n_{i}, respectively) to the real and imaginary parts of the permittivity using the equations

$$\varepsilon_{\mathrm{r}} = n_{\mathrm{r}}^2 - n_{\mathrm{i}}^2$$
$$\varepsilon_{\mathrm{i}} = 2n_{\mathrm{r}}n_{\mathrm{i}}$$

The phase velocity is related to the refractive index through the equation

$$v_{\mathrm{ph}} = \frac{c}{n_{\mathrm{r}}}$$

Assuming $n_{\mathrm{i}} \ll n_{\mathrm{r}}$ and converting angular frequency to vacuum wavelength λ_0, we get the equation

$$\alpha = \frac{4\pi n_{\mathrm{i}}}{\lambda_0}$$

We have assumed that ε_{i} is sufficiently small such that a first-order calculation holds. We can estimate the regime where the first-order calculation is applicable by considering when would ε_{i} be sufficiently small so that the approximation $n_{\mathrm{r}} \approx \sqrt{\varepsilon_{\mathrm{r}}}$ is still good. By inverting the relations between ε_{r}, ε_{i} and n_{r}, n_{i} above and Taylor expanding assuming $\varepsilon_{\mathrm{i}} < \varepsilon_{\mathrm{r}}$, we get the equation

$$n_{\mathrm{r}} = \sqrt{\frac{\sqrt{\varepsilon_{\mathrm{r}}^2 + \varepsilon_{\mathrm{i}}^2} + \varepsilon_{\mathrm{r}}}{2}} = \sqrt{\varepsilon_{\mathrm{r}}}\left[1 + \frac{1}{8}\left(\frac{\varepsilon_{\mathrm{i}}}{\varepsilon_{\mathrm{r}}}\right)^2\right] + O\left(\left(\frac{\varepsilon_{\mathrm{i}}}{\varepsilon_{\mathrm{r}}}\right)^4\right)$$

which gives us the condition that $\frac{1}{8}\left(\frac{\varepsilon_{\mathrm{i}}}{\varepsilon_{\mathrm{r}}}\right)^2 \ll 1$. Outside of this regime, the first-order calculation we use here would break down.

S.6 FDTD simulations

Simulations of the THz fields were performed with the finite-difference time-domain (FDTD) method using the open-source software package MEEP [6]. The LN slab was treated as an infinite slab in the x and y directions with finite extent in the z direction. The long dimension of the slot waveguide was aligned along the x direction. Material dispersion was included by using a Lorentzian susceptibility with parameters taken from literature [7]. The in-plane LN c-axis was set along the y-axis in the simulation. The lactose slab was treated as an infinite slab along the z direction with extent along the x and y axes that matched the slot. The lactose slab was treated as a homogeneous slab with constant refractive index. The imaginary refractive index of lactose was set to 0 for the purposes of calculating the fill fraction. When using the iterative procedure to solve for material parameters described in the main text, the lactose refractive index was updated during each iteration. Absorbing boundary conditions were used to suppress reflections off the edges of the simulation space.

To calculate the fill fractions, we first calculate the electric field profile of the slot waveguide modes. A y-polarized continuous-wave sheet source was placed inside the LN slab 100 µm from the edge of the slot waveguide and the simulation was run for a long time to allow any transient THz fields to decay away. The slot waveguide only supports one propagating mode with even symmetry along the y-axis in the frequency range of interest, so the electric field mode profile was determined simply by taking a slice of the fields along the yz plane many wavelengths away from the source. The fill fraction was then calculated by numerically evaluating the integral

$$f = \frac{\int_{V_{\text{pert}}} \varepsilon|\boldsymbol{E}|^2\, dV}{\int_V \varepsilon|\boldsymbol{E}|^2\, dV}$$

Note that although the source was y-polarized, the slot waveguide mode has electric field components along the x, y, and z directions. All electric field components were used when evaluating the integrals. This procedure was repeated for the range of frequencies that were investigated.

S.7 Effect of the air gap

In the experiment, the sample thickness (40 μm) was slightly smaller than the slot (50 μm wide) in order to facilitate loading. As a result, there is a 10 μm air gap in the slot. In order to investigate the effect of the air gap, we calculated and compared the fill fraction in the following three cases: (1) the 50-μm slot is completely filled, (2) a 40-μm sample is centered within the 50-μm slot, and (3) a 40-μm sample is located at the edge of the slot. The calculated slot fill fractions are listed in Table S1. The sample position has a very small impact on the fill fraction. Furthermore, we see that taking the value for the completely filled slot and multiplying by 0.8 is a good approximation in the case where the exact sample position is unknown.

50-μm slot completely filled	40-μm sample (centered)	40-μm sample (placed at edge)	Value for filled slot multiplied by 0.8 (approximation used in main text)
0.537	0.429	0.422	0.427

Table S1. Calculated slot fill fractions with air gap present. Values for the sample fill fraction calculated at 0.53 THz for various cases of sample loading.

References

1. A. Yariv and P. Yeh. Photonics: Optical Electronics in Modern Communications. (Oxford University Press, 2007).
2. P. Sivarajah *et al*. THz-frequency magnon-phonon-polaritons in the collective strong-coupling regime. *J. Appl. Phys.* **125**, 213103 (2019).
3. V. R. Almeida, Q. Xu, C. A. Barrios, and M. Lipson. Guiding and confining light in void nanostructure. *Opt. Lett.* **29**, 1209-1211 (2004).
4. X. Zhou *et al*. One-step precise machining of terahertz microstructures on chip-scale lithium niobate via laser dispersion engineering. *Mater. Today Phys.* 35, 101102 (2023).
5. J. D. Joannopoulos, S. G. Johnson, J. N. Winn, and R. D. Meade. Photonic Crystals: Molding the Flow of Light (Princeton University Press, 2008).
6. A. F. Oskooi *et al*. MEEP: A flexible free-software package for electromagnetic simulations by the FDTD method. *Comput. Phys. Commun.* **181**, 687–702 (2010).
7. A. S. Barker and R. Loudon. Dielectric Properties and Optical Phonons in $LiNbO_3$. *Phys. Rev.* **158**, 433–445 (1967).
